# An Objective Pain Score for Chronic Pain Clinic Patients

**DOI:** 10.1155/2021/6695741

**Published:** 2021-02-08

**Authors:** Agnes K. Pace, Melanio Bruceta, John Donovan, Sonia J. Vaida, Jill M. Eckert

**Affiliations:** ^1^Department of Anesthesiology & Perioperative Medicine, Penn State Health Milton S. Hershey Medical Center, Hershey, PA, USA; ^2^Division of Pain Medicine, Department of Anesthesiology & Perioperative Medicine, Penn State Health Milton S. Hershey Medical Center, Hershey, PA, USA; ^3^Department of Physical Medicine and Rehabilitation, University of Texas Southwestern Medical Center, Dallas, TX, USA

## Abstract

**Objectives:**

Although numerous studies have looked at the numeric rating scale (NRS) in chronic pain patients and several studies have evaluated objective pain scales, no known studies have assessed an objective pain scale for use in the evaluation of adult chronic pain patients in the outpatient setting. Subjective scales require patients to convert a subjective feeling into a quantitative number. Meanwhile, objective pain scales utilize, for the most part, the patient's behavioral component as observed by the provider in addition to the patient's subjective perception of pain. This study aims to examine the reliability and validity of an objective Chronic Pain Behavioral Pain Scale for Adults (CBPS) as compared to the traditional NRS.

**Methods:**

In this cross-sectional study, patients were assessed before and after an interventional pain procedure by a researcher and a nurse using the CBPS and the NRS. Interrater reliability, concurrent validity, and construct validity were analyzed.

**Results:**

Interrater reliability revealed a fair-good agreement between the nurse's and researcher's CBPS scores, weighted kappa values of 0.59 and 0.65, preprocedure and postprocedure, respectively. Concurrent validity showed low positive correlation for the preprocedure measurements, 0.34 (95% CI 0.16–0.50) and 0.47 (95% CI 0.31–0.61), and moderate positive correlation for the postprocedure measurements, 0.68 (95% CI 0.56–0.77) and 0.67 (95% CI 0.55–0.77), for the nurses and researchers, respectively. Construct validity demonstrated an equally average significant reduction in pain from preprocedure to postprocedure, CBPS and NRS median (IQR) scores preprocedure (4 (2–6) and 6 (4–8)) and postprocedure (1 (0–2) and 3 (0–5)), *p* < 0.001. *Discussion*. The CBPS has been shown to have interrater reliability, concurrent validity, and construct validity. However, further testing is needed to show its potential benefits over other pain scales and its effectiveness in treating patients with chronic pain over a long-term. This study was registered with ClinicalTrial.gov with National Clinical Trial Number NCT02882971.

## 1. Introduction

Pain self-report continues to be considered the gold standard of pain assessment [1]. A number of self-reporting pain assessment tools have been designed and validated in patients with chronic pain, including the most commonly used numeric rating scale (NRS), the verbal rating scale (VRS), and the visual analog scale (VAS) [2–6]. These scales require patients to place a quantitative rating on their pain sensation and convert a subjective feeling into a quantitative number for pain measurement and long-term evaluation over time. While these scales may place an appropriate emphasis on the patient, unfortunately, they allow for bias and lack of objectivity due to the data being completely patient-reported.

Chronic pain patients are asked to rate their pain using the NRS or the VAS at many points during their care. Patient-reported pain scores are valid in the moment the scores are obtained but are unidimensional and do not account for the impact chronic pain has on a patient's daily life [7]. For this reason, multidimensional pain scoring systems have been developed to allow patients to better qualify their pain with standardized descriptive terms, measure the pain impact on their daily activities, and improvement in these activities after pain interventions [1]. However, these scoring systems have limitations, one of which is relying on a patient's memory to measure a change in pain over time.

Observational pain scoring systems such as the Critical Care Pain Observation Tool (CPOT) and the behavioral pain scale (BPS) have been created and validated in populations of patients who are unable to report their pain level, such as children, critically ill, intellectually disabled, and dementia patients [8–12]. In these patient populations, clinicians must use observer-objective criteria because self-report is not possible or reliable. Behavioral pain scoring systems have been developed to allow for the establishment of independent measures of a patient's pain that can be compared over time in inpatient settings, where observer-based scoring systems must be implemented [9, 13–15]. However, these scales have not been validated in other populations such as chronic pain patients.

In order to be clinically valuable, a pain scoring system has to be simple and must yield congruent results across different types of healthcare providers, while retaining its validity as a measure of the patient's pain [16]. Currently, there is no observational pain scale for use in the adult outpatient setting. An observational pain scale can allow for objective pain measurement over time and immediately after treatment without the bias associated with the self-report system. For this reason, the purpose of this study is to introduce and validate a behavioral pain scale in the adult outpatient chronic pain setting. This scale has the potential to allow for an objective pain measurement that is reliable across multiple raters and comparable over time, which can help in judging the success of pain treatments.

## 2. Materials and Methods

### 2.1. Subjects and Setting

The Human Subject Protections Office at our institution approved this prospective observational study on July 7th, 2016 (ID: STUDY00003685), and it was performed between September 2016 and December 2017. This study was registered with ClinicalTrial.gov with National Clinical Trial Number NCT02882971. Patients' oral and written consent was obtained before data collection during their first clinical visit. One hundred and one patients were seen at the Penn State Milton S. Hershey Medical Center' Chronic Pain Management Clinic. Patients less than 18 and older than 75 years of age, pregnant and lactating women, patients with chronic malignant pain, cognitive impairment, those unable to self-report pain, those with uncontrolled psychiatric conditions, uncontrolled substance abuse, and lost to follow-up were excluded. Inclusion criteria were adults 18–75 years of age who were able to self-report pain levels using a validated pain scale and fluent in spoken English.

### 2.2. Materials

#### 2.2.1. Chronic Pain Behavioral Pain Scale for Adults

There are three well-known and previously tested behavioral pain scales, the Princess Margaret Hospital Pain Assessment Tool, the Children's Hospital of Eastern Ontario Pain Scale (CHEOPS), and the Behavioral Observational Pain Scale (BOPS). These scales were primarily designed to assess postoperative pain in the pediatric population and are described in detail elsewhere [8]. Briefly, observational categories of pain behaviors such as facial expression, vocalization, and body position were recorded and graded, giving rise to a number scale ranging from no pain to the worst pain. We created the CBPS to consist of five categories: pain bother, anxiety, face, activity, and interaction (Table 1). Each category was graded from 0 to 2 creating a total summative minimum value of 0 (no pain) and maximum value of 10 (the worst pain). The first category involved asking the patient a question rather than being observational, while the other four categories were solely observational. The severity range follows the traditional classification found in the NRS and FLACC pain scale of mild (1–3), moderate (4–6), and severe pain (7–10).

#### 2.2.2. Numeric Rating Scale

The numeric rating scale (NRS) has been validated and widely implemented across the healthcare industry for evaluation of acute and chronic pain and for various pain types [17]. It involves the healthcare provider asking the patient to rate the intensity of their current pain on a scale of 0 (“no pain”) to 10 (“worst possible pain”), resulting in that patient's pain score.

### 2.3. Study Procedures

Potential study subjects were identified on their arrival to the chronic pain management center for interventional pain procedures. A prior evaluation by a pain management physician had determined that an interventional pain procedure was the best choice for chronic pain relief. For training purposes, the assigned nurse and research team member were provided a copy of the Chronic Pain Behavioral Pain Scale for Adults.

Each team member was individually instructed by the principal investigator in the use of the scale immediately preceding their first time use. This training included a discussion of the CBPS five categories and the use of the scoring system. Medical record information collected included gender, age, ethnicity, procedure performed, and research team member performing the scales. The study consisted of three parts to assess the interrater reliability, concurrent validity, and construct validity for the CBPS.

#### 2.3.1. Interrater Reliability

This part of the study aimed to determine whether there was homogeneity and consensus between different healthcare providers evaluating the same patient. It consisted of two trained observers independently assessing the patient's pain level by following the CBPS instructions. The two observers consisted of the patient's assigned nurse and a member of the research team. Scoring was performed at the same time the NRS was done: during the initial nursing check-in process and at the postintervention check-out process. The two trained observers were simultaneously in the room with the patient, watching the event (check-in and check-out) at the same time, independently recording patient-provided answers for the NRS score and category 1 of the CBPS. Then, they independently assessed the patient and recorded observations for CBPS categories 2–5. A total of eight nurses were paired to three researchers in collecting the data.

#### 2.3.2. Concurrent Validity

The NRS was chosen as the standard measure for testing the concurrent validity of the CBPS. The purpose of this part of the study was to determine whether the CBPS correlates with the previously validated and widely used NRS on the present criterion (pain). As previously mentioned, both scales were used preprocedure and postprocedure by the same two observers, independently of each other.

#### 2.3.3. Construct Validity

In construct validity, we compared the CBPS results obtained before and after the interventional pain procedure. The purpose of this part of the study was to determine whether the CBPS is indeed measuring the construct (pain) and providing a significant and reliable difference between the preintervention and postintervention pain score.

### 2.4. Statistical Analysis

#### 2.4.1. Interrater Reliability

Weighted kappa evaluation was used to determine interrater reliability using SAS version 9.4, SAS Institute Inc., Cary NC, copyright 2002–2012. The kappa statistic is an index of agreement (Kw) with values ranging between 0 and 1, where <0.55 is an unsatisfactory agreement, ≥0.55 is fair, ≥0.65 is good, and ≥0.77 is excellent [18].

#### 2.4.2. Concurrent Validity

The correlation between NRS and CBPS was analyzed using the nonparametric Spearman rank order correlation coefficient with *p* < 0.05 considered statistically significant. First, correlation was calculated using preprocedure data and whether it was collected by a nurse or a researcher. Second, correlation was calculated using postprocedure data and whether it was collected by a nurse or a researcher.

#### 2.4.3. Construct Validity

First, nonparametric tests were used to determine whether there was a significant difference at the preprocedure and postprocedure timepoints, for both nurse and researcher, respectively, then combined. Second, nonparametric tests were used to determine whether there was a significant change from preprocedure to postprocedure obtained scores, for both nurse and researcher, respectively, then combined. Values were reported in median and interquartile range (IQR). Wilcoxon's signed ranks test was used to compare the effect of intervention on both NRS and CBPS, with *p* < 0.01 taken as significant. Spearman rank order correlation statistics were then used to determine whether the change in behavioral pain score correlated with the change in the numeric pain score for nurses and researchers, respectively, and then combined.

## 3. Results

A total of 101 evaluations were performed. We could not obtain postprocedure data for 2 of these evaluations due to unperformed procedures (one patient being ill and the other having no driver). The researcher group consisted of two medical students and an anesthesiology resident, whereas the nurse group consisted of 8 chronic pain nurses.

The patient population is described in Table 2. Trigger point injection (TPI) sites were not recorded. Epidural steroid injection (ESI) sites (# of cases) were recorded as lumbar (1), caudal (2), and L5-S1 level (1). Transforaminal epidural steroid injection (TFESI) sites included L5-S1 level (3), L4-L5 level (3), and L3-L4 + L4-L5 levels (1). Peripheral nerve blocks included ulnar (1), radial (1), medial (1), occipital (7), genicular (3), intercostal (3), superficial peroneal (1), suprascapular (1), and ilioinguinal + iliohypogastric (1) nerves. Facet joint injection sites included C1-C2 (1), C6-C7 (1), L3-L4 (2), L4-L5 (1), L4-5 + L5-S1 (5), and lumbar (1) facet joints. Bursa injection sites included the hip (5) and nonrecorded (1). Median branch nerve blocks were documented as C2, C3, C4 (1), L3, L4, L5 (2), and L4, L5 (1) nerves. Peripheral nerve cryoablations included occipital nerve (1) and genitofemoral nerve (1).

### 3.1. Interrater Reliability

**Figure 1:**
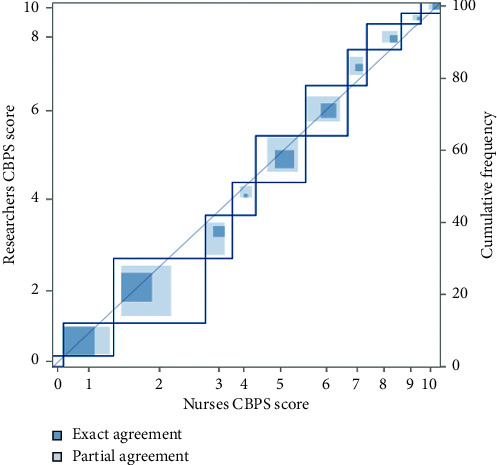
Interrater reliability for CBPS scores between nurses and researchers preprocedure.

**Figure 2:**
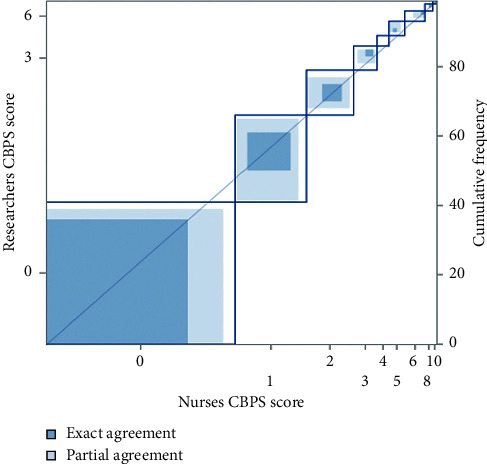
Interrater reliability for CBPS scores between nurses and researchers postprocedure.

To determine interrater reliability, 101 paired observations were made preprocedure, and 99 paired observations were made postprocedure. There was a fair-good agreement between the nurses' and researchers' CBPS scores, with overall weighted kappa (Kw) values of 0.59 (35.6%) and 0.65 (58.6%) preprocedure and postprocedure, respectively (Figures 1 and 2). Broken down further, the Kw values preprocedure and postprocedure, respectively, were 0.49 (62.4%) and 0.52 (81.8%) for anxiety, 0.54 (66.3%) and 0.65 (88.9%) for face, 0.58 (70.3%) and 0.38 (68.7%) for activity, and 0.68 (80.2%) and 0.51 (87.9%) for interaction.

### 3.2. Concurrent Validity

The correlation coefficients for the preprocedure measurements were 0.34 (95% CI 0.16–0.50) and 0.47 (95% CI 0.31–0.61) for the nurses and researchers, respectively. These values reflect low positive correlation (*p*=0.053 and *p*=0.737, respectively). The correlation coefficients were higher for the postprocedure measurements for both nurses (0.68 with 95% CI 0.56–0.77) and researchers (0.67 with 95% CI 0.55–0.77). These values reflect a moderate positive correlation (*p*=0.006 and *p*=0.009, respectively).

### 3.3. Construct Validity

The CBPS score before the procedure was higher (with a median (IQR) score of 4 (2–6)) than the CBPS score after the procedure (1 (0–2)) for both nurses and researchers, respectively, and combined. The NRS score before the procedure was higher (with a median (IQR) score of 6 (4–8)) than the NRS score after the procedure (3 (0–5)) for both nurses and researchers, respectively, combined.

The median CBPS and NRS for both nurses and researchers, respectively, were significantly different from each other for both preprocedure and postprocedure, *p* < 0.001. This remained the same when nurses and researchers were combined.

There was a statistically significant median reduction in both CBPS and NRS, *p* < 0.001.

The correlation coefficients for the change in CBPS in comparison to the change in NRS were 0.43 (95% CI 0.25–0.58) and 0.48 (95% CI 0.32–0.62) for the nurses and researchers, respectively. These values reflect low positive correlation. When both nurses and researchers were combined, the correlation coefficient for the change in CBPS in comparison to the change in NRS was 0.46 (95% CI 0.34–0.56). These values reflect low positive correlation as well.

## 4. Discussion

The CBPS that we devised is a simple pain scale that can be quickly and easily used to assess a patient's current chronic pain status from a multidimensional aspect, with the hope of better pain management. It does not rely on self-recall of the patient and thus may be beneficial in decreasing the potential overtreatment of pain. This is especially important given the current opioid epidemic and increased number of deaths related to opioid overdose, as recently reported by the Center for Disease, Control, and Prevention, 2018 Annual Surveillance Report of Drug-Related Risks and Outcomes [19].

### 4.1. Interrater Reliability

Overall, the interrater reliability for CBPS scoring showed moderate agreement, with a higher agreement score for postprocedure scores. The interrater reliability was similar across the board for all categories. Our values are on the lower to middle end of other behavioral pain scale studies such as the Face, Legs, Activity, Cry, Consolability (FLACC) pain assessment tool [20], the Critical Care Pain Observational Tool (CPOT) [21], and the Behavioral Observational Pain Scale (BOPS) [8].

A possible reason for lower interrater reliability in our study is the discrepancy between groups (nurse vs researcher) in experience working with chronic pain patients. The difference in experience and exposure to this patient population could play a role in the differential scoring of behavioral observations. In particular, it is noted that in the patients with the largest differences in pain scores, the researchers set a higher pain score than the nurses in almost all cases. It suspected that with more experienced practitioners doing the scoring instead of researchers with varied but relatively limited exposure to chronic pain patients, there would be an evening out of the scores. Previous studies have noted differences in clinical training, specifically between physicians and nurses, with nurses both having spent more time with patients and having more training on how to perceive the discomfort of patients [22].

Furthermore, while not collected during the study, it was noted through observation that a large percentage of the pain patients in this study had been seen multiple times in the pain clinic even if the procedures themselves were new procedures for the patient. With long-term management of patient care, there is increased communication and openness of patients with the care provider which could allow for a different impression of the patient [23]. It is suspected that some of the differences in the CBPS are from the nurses having seen the patients in a prior visit and having a better understanding and comfort level with the patient in the study.

### 4.2. Concurrent Validity

There was a low positive correlation between the NRS and the CBPS preprocedure and a moderate positive correlation postprocedure. The low positive correlation was expected, as the reason for development of the CBPS was to improve upon the potential self-bias and overexaggerating of the NRS. It is not surprising that the NRS would likely show a higher initial pain score compared to the CBPS, as patients are presenting for an interventional pain procedure at the time of evaluation and, hence, are more likely to be thinking about their pain as more bothersome at that moment [24–26].

On the other hand, we realize there is some fallacy in using the NRS as the gold standard against which we are comparing the CBPS, when in actuality, we are trying to improve upon the NRS. Choosing a behavioral pain scale that has been validated for use in adult outpatient clinic settings for our gold standard would have been ideal; however, there are currently no pain scales we know that meet this criterion. Although choosing a different adult behavioral pain scale, such as the Pain Assessment in Advanced Dementia (PAINAD) Scale or the CPOT, may have been an option, we theorized that the use of these scales in an outpatient clinic setting would also have its limitations [27]. For example, none of our outpatient clinic patients would be on a mechanical ventilator, and the absence of movement in these patients should not lead to a lower pain score (as would be seen with use of CPOT). Similarly, with the use of the PAINAD scale, the breathing subcategory is not applicable to the outpatient setting, since these patients have been dealing with their pain for a significant period of time with no acute changes requiring hyperventilation or labored breathing.

The potential usefulness of the CBPS in assessing chronic pain over the typical time course of years and thus multiple physician visits was not able to be tested in this study. It is suspected that the behavioral pain scale would be able to chronicle the effectiveness of a treatment option as previous studies have noted the effectiveness of using observational components to track pain in particular facial expressions and the ability to detect both mimicry of pain and the change in pain over a period of time [28, 29]. Furthermore, this study would allow for the reduction of the ability of patients with an incentive to exaggerate reported pain for litigation, workman's compensation, self-esteem, and opioids [24–26].

### 4.3. Construct Validity

Patients involved in this study were evaluated and treated with various interventional pain procedures. The intervention offered to each patient was determined by the expertise of our pain physicians, as deemed the best option for their chronic pain control based on history and physical examination.

For this study, the assumption was made that each patient's pain was highest immediately prior to the procedure, and per daily practice, was obtained using the standard NRS. It has been documented that patients tend to exaggerate pain complaints, especially in those with multiple comorbidities [30].

In addition, the unidimensional capability of the NRS and its reliance on memory may at least partially explain the higher NRS preprocedure score compared to the preprocedure CBPS. Nonetheless, the results of the nonparametric tests in the construct validity section indicate that both the NRS and the CBPS demonstrate an average reduction in pain from preprocedure to postprocedure of approximately 3 (*p* values <0.001). In other words, like the NRS, the CBPS score was significantly lower after the interventional pain treatment, suggesting that the CBPS does indeed assess for pain.

We looked at our raw data even further after our initial results to determine whether the change in score between the preprocedure and postprocedure CBPS scores was mainly due to the one subjective, patient-reported subcategory of our CBPS—pain bother (“How much is your pain bothering you now?”). Analysis showed that the difference in postprocedure CBPS was exactly the same as the difference in response to pain bother in 43 or 43.3% of our patients. Although this finding shows that the subjective component of our otherwise objective pain scale significantly contributed to our positive results, the other objective components also played as large a role, further validating that our pain scale does assess for pain.

### 4.4. Study Limitations

There are several limitations to this study. First, this is a single center study where the patients included underwent a wide range of interventional pain procedures, with some procedures providing much faster onset of pain relief compared to others. The inclusion of different interventional pain procedures suggests treatment of different types of pain with varying etiologies. This could affect both our preprocedure and postprocedure data. For example, a patient presenting with chronic myofascial pain in his neck and upper back may not necessarily have as much limitation in activity as a patient with chronic low back pain with radiculopathy. Related to this difficulty, since this study included patients that experienced a wide variety of pain conditions with only one source of pain being addressed with the procedure, it can be difficult to assess how much a patient's gait would improve after receiving treatment for chronic upper extremity myofascial pain if underlying lower extremity pathology was also present. In addition, we did not include a criteria for choosing each intervention as they were chosen randomly, which may have played a role in lower preprocedural pain scores. Our study includes 101 patients with a wide variety of procedures which can potentially lead to a lower statistical power. However, the numbers are sufficient to support some conclusions because no prior studies have assessed a behavioral pain scale on chronic pain patients.

Another limitation is the experience of each practitioner. Since the CBPS is a behavioral scale that relies on the practitioner to use clinical judgment, this can allow for some variations in interpretation dependent on the practitioner performing the assessment. Although defined measures were outlined and each practitioner received verbal guidance prior to beginning the study, any assessments involving behavioral indicators will inevitably allow for even slight variations in interpretation which could become magnified when there are only three options for each category. The CBPS would likely give the best correlation when used by experienced chronic pain health professionals. However, the CBPS is a simple scale that, with the appropriate training, could be used by any group of health professionals.

The patient population involved in this study was highly homogeneous, with 88% of the population identifying themselves as Caucasian as well as all patients being fluent in English. These facts, along with the small sample size of this study, make it difficult to apply this pain scale globally, as it has been extensively documented in the literature that patients of different ethnicities both report and physically exhibit pain in different ways and with different facial expressions [31–33]. In addition, prior studies have demonstrated the difficulty of different racial groups of interpreting the facial expressions of people of a different race [34, 35]. We also excluded patients who are in need for an objective pain scale, such as those with chronic malignant pain, cognitive impairment, and those unable to self-report pain for various other reasons, including young children. Further studies would be needed to validate the use of this pain scale for evaluation of these specific populations.

Finally, as mentioned above, using the NRS to compare our CBPS scoring system is a limitation in itself, as one of the goals of developing this pain scale is to improve upon the NRS. In addition, the difference between preprocedure and postprocedure CBPS scores was exactly the same as the difference between preprocedure and postprocedure pain bother scores for 43% of our patients, suggesting that the one subjective measurement of our pain scale played a large role in the significant difference between preprocedure and postprocedure scores. Perhaps, an ideal scale needs to include both subjective as well as objective assessments of pain, in order to more comprehensively encompass the multifaceted and complex etiology of a person's pain sensation. It was documented more than 50 years ago that “pain is what the person says it is and exists whenever he or she says it does” [36]. As we have come to accept that patients are the experts in their pain experience, it seems that subjective reporting cannot be excluded if we are to accurately and effectively treat pain.

## 5. Conclusion

The CBPS was found to be easy to use, with no inconvenience to the patients and minimal additional time spent to administer it in a busy outpatient pain clinic. Given our small sample size and homogeneous patient population, additional studies would need to be performed to further validate the use of this scale as an objective measure of pain, especially in patients of non-Caucasian background. Despite its limitations, use of the CBPS may allow chronic pain practitioners to better assess, over the course of a chronic pain patient's treatment, the potential improvements seen in the patient's pain management.

## Figures and Tables

**Table 1:** Chronic pain behavioral pain scale for adults (CBPS).

Category	Score	0	1	2
1	Pain bother “How much is your pain bothering you right now?”	A little bit	Somewhat	Quite a bit or very much

2	Anxiety	Relaxed and content	Relaxes with reassurance	Difficult or unable to comfort

3	Face	Smile or neutral	Neutral with occasional grimace or frown	Frowning or grimace most of the time

4	Activity	Normal posture and moves easily	Favoring posture and movement is somewhat limited by pain	Tense and guarded with little movement or frequent position changes

5	Interaction	Calm with normal conversation	Uneasy with hesitant speech and some complaints of pain	Agitated or crying with rapid speech and mostly complaints of pain

**Table 2:** Demographics and type of pain management procedure.

Characteristics	*n*
Male (%)	36 (35.6)
Female (%)	65 (64.4)
Age (years) (mean ± SD)	52.2 ± 13.9

Ethnicity (%)	
Caucasian	89 (88.1)
African American	10 (9.9)
Hispanic or Latino	2 (2.0)

Type of procedure (%)	
Trigger point injection (TPI)	32 (31.7)
Epidural steroid injection (ESI)	4 (4.0)
Transforaminal epidural steroid injection (TFESI)	7 (6.9)
Interlaminar ESI (L4-L5)	1 (1.0)
Peripheral nerve block	19 (18.8)
Facet joint injection	11 (10.9)
Bursa injection	6 (5.9)
Median branch block	4 (4.0)
SI join injection	3 (3.0)
SI ligament injection	2 (2.0)
TPI + occipital nerve block	2 (2.0)
TPI + bursa injection (shoulder)	1 (1.0)
Peripheral nerve cryoablation	2 (2.0)
Stellate ganglion block	1 (1.0)
TFESI (L4-L5) + facet joint injection	1 (1.0)
Intraarticular hip injection	2 (2.0)
Scar neuroma injection	1 (1.0)
Piriformis injection	1 (1.0)
Sacrococcygeal joint injection	1 (1.0)

Data presented as percentage (%) or mean ± standard deviation (SD).

## Data Availability

The data used to support the findings of this study are included within the article.
